# The NOTCH3 Downstream Target HEYL Is Required for Efficient Human Airway Basal Cell Differentiation

**DOI:** 10.3390/cells10113215

**Published:** 2021-11-18

**Authors:** Manish Bodas, Bharathiraja Subramaniyan, Andrew R. Moore, Jordan P. Metcalf, Sarah R. Ocañas, Willard M. Freeman, Constantin Georgescu, Jonathan D. Wren, Matthew S. Walters

**Affiliations:** 1 Department of Medicine, Section of Pulmonary, Critical Care & Sleep Medicine, University of Oklahoma Health Sciences Center, Oklahoma City, OK 73104, USA; manish-bodas@ouhsc.edu (M.B.); Bharathiraja-Subramaniyan@ouhsc.edu (B.S.); Andrew-R-Moore@ouhsc.edu (A.R.M.); jordan-metcalf@ouhsc.edu (J.P.M.); 2 Oklahoma City Veterans Affairs Medical Center, Oklahoma City, OK 73104, USA; bill-freeman@omrf.org; 3 Department of Physiology, University of Oklahoma Health Sciences Center, Oklahoma City, OK 73104, USA; Sarah-Ocanas@ouhsc.edu; 4 Genes & Human Disease Research Program, Oklahoma Medical Research Foundation, Oklahoma City, OK 73104, USA; Constantin-Georgescu@omrf.org (C.G.); Jonathan-Wren@omrf.org (J.D.W.)

**Keywords:** NOTCH3 signaling, HEYL, proliferation, differentiation, airway epithelium, basal stem/progenitor cells, club cells, goblet cells, ciliated cells, COPD

## Abstract

Basal cells (BCs) are stem/progenitor cells of the mucociliary airway epithelium, and their differentiation is orchestrated by the NOTCH signaling pathway. NOTCH3 receptor signaling regulates BC to club cell differentiation; however, the downstream responses that regulate this process are unknown. Overexpression of the active NOTCH3 intracellular domain (NICD3) in primary human bronchial epithelial cells (HBECs) on in vitro air–liquid interface culture promoted club cell differentiation. Bulk RNA-seq analysis identified 692 NICD3-responsive genes, including the classical NOTCH target HEYL, which increased in response to NICD3 and positively correlated with SCGB1A1 (club cell marker) expression. siRNA knockdown of HEYL decreased tight junction formation and cell proliferation. Further, HEYL knockdown reduced club, goblet and ciliated cell differentiation. In addition, we observed decreased expression of HEYL in HBECs from donors with chronic obstructive pulmonary disease (COPD) vs. normal donors which correlates with the impaired differentiation capacity of COPD cells. Finally, overexpression of HEYL in COPD HBECs promoted differentiation into club, goblet and ciliated cells, suggesting the impaired capacity of COPD cells to generate a normal airway epithelium is a reversible phenotype that can be regulated by HEYL. Overall, our data identify the NOTCH3 downstream target HEYL as a key regulator of airway epithelial differentiation.

## 1. Introduction

The mucociliary epithelium is a multicellular tissue that lines the conducting airways and functions as a barrier to protect the lung from environmental insults [1,2,3,4]. Basal cells (BCs) are the resident stem/progenitor cells of the mucociliary airway epithelium in humans and mice that initiate repair of the epithelium during homeostasis and following injury [1,2,3,4,5,6,7,8,9]. Initiation of repair leads to increased BC proliferation to maintain the stem cell compartment via self-renewal and to generate intermediate luminal cell progenitors (also termed basal-intermediate, supra-BCs or secretory primed BCs). In turn, intermediate cells differentiate into club cells which function as progenitors for goblet and ciliated cells [5,6,7,8,9]. Alterations in the ratio of differentiated cell types (defined as epithelial remodeling), including a reduction in the number of club cells, lead to impaired host barrier function and are associated with chronic lung diseases, including asthma, idiopathic pulmonary fibrosis (IPF) and chronic obstructive pulmonary disease (COPD) [2,10,11,12,13]. Therefore, identifying the mechanisms that regulate BC proliferation and differentiation are central to understanding the pathophysiology of chronic lung disease and the lung’s response to injury.

The NOTCH signaling pathway regulates BC stem/progenitor function and cell fate decisions in the human and murine mucociliary airway epithelium [5,14,15,16,17,18,19,20,21,22,23,24,25,26,27,28,29,30,31,32,33,34,35,36,37,38,39,40,41,42,43,44,45,46]. Canonical NOTCH signaling is initiated by the binding of a ligand (DLL1, DLL3, DLL4, JAG1 or JAG2) to one of the four receptors (NOTCH1–4) located on the surface of a neighboring cell. This leads to proteolytic cleavage of the receptor and release of the NOTCH intracellular domain (NICD) into the cytoplasm [47,48]. The NICD then translocates to the nucleus and regulates the expression of multiple downstream genes [47,48]. Despite the knowledge that activation of NOTCH3 signaling is critical for regulating BC proliferation and club cell differentiation [5,19,27], the downstream genes and pathways that regulate this process are unknown. The present study was designed to address this gap in our knowledge. Using the in vitro air–liquid interface (ALI) system to mimic the human mucociliary airway epithelium, we have characterized the NOTCH3-dependent downstream genes/pathways in differentiating primary human bronchial epithelial cells (HBECs) and identified that HEYL, a known NOTCH3 target gene, regulates their proliferation and differentiation into club, goblet and ciliated cells. In addition, comparison of HBECs from COPD vs. normal donors demonstrated that expression of HEYL is reduced in COPD cells and correlates with the impaired capacity of COPD-derived HBECs to differentiate into a normal mucociliary epithelium. Furthermore, lentivirus-mediated overexpression of HEYL in COPD HBECs promoted differentiation into club, goblet and ciliated cells. Combined, these data suggest the impaired capacity of COPD HBECs to generate a normal airway epithelium in vitro is a reversible phenotype that can be regulated by HEYL.

## 2. Materials and Methods

### 2.1. Primary Human Bronchial Epithelial Cell (HBEC) Culture

Primary HBECs from normal donors (nonsmokers and smokers) and COPD smokers were purchased commercially (catalog number CC-2540 and 00195275, Lonza, Morristown, NJ, USA) and cultured as described [14]. In total, *n* = 18 cell donors were used in this study (Table 1).

### 2.2. Air–Liquid Interface (ALI) Culture

HBECs were differentiated into a mucociliary airway epithelium using ALI culture for up to 28 days as described [14].

### 2.3. RNA Extraction, cDNA Synthesis and Quantitative PCR Analysis

RNA extractions, cDNA synthesis and quantitative PCR (qPCR) analysis were performed as described previously [14]. For qPCR, all samples were analyzed in duplicate with relative expression levels determined using the dCt method with actin beta (ACTB) as the endogenous control. The following PrimePCR gene-specific primers were purchased from Bio-Rad: ACTB (qHsaCED0036269), SCGB1A1 (qHsaCID0018013), KRT5 (qHsaCED0047798), MUC5AC (qHsaCID0017663), FOXJ1 (qHsaCID0016777), NOTCH3 (qHsaCID0006529), HEYL (qHsaCID0006092), CLDN3 (qHsaCEP0032466), OCLN (qHsaCEP0041012), TJP1 (qHsaCIP0031627), TJP2 (qHsaCIP0026125), TJP3 (qHsaCIP0030881), PARD3 (qHsaCIP0039350), PARD6B (qHsaCEP0051277) and MKI67 (qHsaCID0011882). The assays were performed using the manufacturer’s recommended cycling parameters.

### 2.4. Single-Cell RNA Sequencing (scRNA-Seq)

HBECs from a single normal nonsmoker donor were cultured on ALI for 9 days and harvested for scRNA-seq analysis. To generate single-cell suspensions, the cells were trypsinized for 3 min and following neutralization passed through a Flowmi tip strainer (40 µm porosity, catalog number H13680-0040, SP Bel-Art, Wayne, NJ, USA). Cells were counted on a hemocytometer prior to diluting them to 800 cells/µL in 0.1% BSA/PBS buffer for scRNA-seq library preparation with Chromium Single Cell 3′ Reagent Kits v3 (catalog number PN-1000075, 10X Genomics, Pleasanton, CA, USA). scRNA-seq libraries were generated according to 10X Genomics User Guide CG000183 Rev C. Briefly, a reaction mix containing 12,800 cells was loaded into a Chromium Chip B, targeting cell recovery of 8000 cells. After generating gel beads-in-emulsion (GEMS) on the Chromium controller, GEMS were transferred to PCR tubes (catalog number 951010022, Eppendorf, Hamburg, Germany) for GEM reverse transcription (GEM-RT). After cleanup with Dynabeads MyOne Silane (catalog number PN-2000048, 10X Genomics), cDNA was amplified with 11 cycles. Amplified cDNA was cleaned with 0.6X SPRISelect reagent (catalog number B23318, Beckman Coulter, Pasadena, CA, USA). Cleaned cDNA was then quality checked on an Agilent Tapestation 4150 (catalog number G2992AA, Agilent, Santa Clara, CA, USA) using a High Sensitivity D5000 ScreenTape (catalog number 5067-5592, Agilent) and quantified using Qubit dsDNA High Sensitivity Assay Kit (catalog number Q32851, Thermo Fisher Scientific, Waltham, MA, USA) read on a Qubit 4 Fluorometer (catalog number Q33238, Thermo Fisher Scientific). An aliquot of 25% of the cleaned cDNA was used for library construction (fragmentation, end repair, A-tailing, adaptor ligation), according to the manufacturer’s instructions. Libraries were indexed (catalog number PN-220103, 10X Genomics) using 10 cycles of PCR. Amplified libraries were cleaned with SPRISelect reagent using a double-sided size selection protocol. Cleaned libraries were quality checked on an Agilent Tapestation 4150 using a High Sensitivity D1000 ScreenTape (catalog number 5067-5584, Agilent) and quantified using Qubit dsDNA High Sensitivity Assay Kit. Libraries were diluted to 4 nM prior to sequencing on a NovaSeq 6000 S1 flow cell with 28 cycles for read 1 and 90 cycles for read 2. Cellranger v3.1.0 (10X Genomics) cellranger mkfastq was used to demultiplex fastq files from raw base call (BCL) files. Fastq files were then aligned to Homo sapiens genome assembly GRCh38-3.0.0 (hg38) and filtered, and barcodes/UMIs were counted using the cellranger count function. Summary metrics revealed 11,567 cells with an average of 8452 reads/cell and a median of 1382 genes/cell. scRNA-seq data were visualized using Loupe Cell Browser (10X Genomics).

### 2.5. Lentivirus-Based Overexpression of NICD3 and HEYL

Generation of control (empty vector)-, NICD3- or HEYL-expressing replication-deficient lentiviruses was performed as described [14]. Briefly, cells were infected with each virus at a multiplicity of infection (MOI) of 50 (5 × 10^6^ viral genomes/1 × 10^5^ cells) at the time of seeding the cells on ALI to ensure that >90% of cells were infected (GFP^+^) with each virus as described [14]. The HEYL-expressing lentivirus (catalog number RC202851L1) and its empty vector control (catalog number PS100064) were purchased from OriGene (Rockville, MD, USA).

### 2.6. Bulk RNA Sequencing (Bulk RNA-Seq)

Lenti-Control or Lenti-NICD3-transduced cells (*n* = 6 normal nonsmoker donors) were harvested as a function of time on ALI (days 0, 3, 5 and 7), and the genome-wide transcriptome changes in response to NICD3 expression were assessed by bulk RNA-seq. Total RNA was extracted from each sample (described above) and bulk RNA-seq was performed on a NextSeq 500 Flowcell, High SR75 (Illumina, San Diego, CA, USA), following library preparation using the QuantSeq 3′ mRNA-Seq Library Prep Kit FWD for Illumina (Lexogen, Vienna, Austria). RNA-seq data processing followed the guidelines and practices of the ENCODE and modENCODE consortia regarding proper experimental replication, sequencing depth, data and metadata reporting and data quality assessment (https://www.encodeproject.org/documents/cede0cbe-d324-4ce7-ace4-f0c3eddf5972/, accessed on 22 April 2019). Raw sequencing reads (in a FASTQ format) were trimmed of residual adaptor sequences using Scythe software. Low-quality bases at the beginning or the end of sequencing reads were removed using sickle, and then the quality of the remaining reads was confirmed with FastQC. Further processing of quality sequencing reads was performed with utilities provided by the Tuxedo Suite software. Reads were aligned to the Homo sapiens genome reference (GRCh38/hg38) using the TopHat component, and then cuffquant and cuffdiff were utilized for gene-level read counting and differential expression analysis. Genes that were significantly differentially expressed in response to NICD3 for at least one time point were determined using a threshold on the false discovery rate (FDR)-adjusted *p*-value of 0.05 (FDR-adjusted *p* < 0.05). Ingenuity Pathway Analysis (IPA) (Qiagen, Redwood City, CA, USA) was used to identify molecular pathways altered in response to NICD3 using an unrestricted analysis.

### 2.7. siRNA-Mediated Knockdown of NOTCH3 and HEYL

HBECs were transfected with either 1 pmol of Silencer Select Negative Control No. 1 siRNA (catalog number 4390844), NOTCH3 siRNA (catalog number 4392420; assay ID s453556) or HEYL siRNA (catalog number 4392420; assay ID s223702) (all from Thermo Fisher Scientific) as described previously [14].

### 2.8. Immunofluorescence Staining

Immunofluorescent staining of either differentiating cells in ALI wells or paraffin-embedded sections of ALI wells, human bronchus from healthy nonsmokers (Donor 1: age 27, female; Donor 2: age 40, female; and Donor 3: age 27, female; catalog number HuFPT111, US Biomax, Inc., Rockville, MD, USA) or mouse trachea (C57BL/6, Normal, Female, 12 weeks) was performed as described [14]. For mouse trachea, the sections were subjected to an additional 1 h of blocking at room temperature, with 20 µg/mL Affinipure Fab fragment goat anti-mouse IgG (H + L) (catalog number 115-007-003, Jackson ImmunoResearch Laboratories, Inc. West Grove, PA, USA). The following primary antibodies were used: HEYL (5 µg/mL, catalog number H00026508-M03, Abnova, Taipei, Taiwan), SCGB1A1 (5 µg/mL, catalog number RD181022220-01, BioVendor LLC, Asheville, NC, USA), KRT5 (2 µg/mL, catalog number PA1-37974, Thermo Fisher Scientific), KRT8 (5 µg/mL, catalog number NBP2-16094, Novus Biologicals, Centennial, CO, USA), MUC5AC (1.4 µg/mL, catalog number MA5-12178, Thermo Fisher Scientific), FOXJ1 (10 µg/mL, catalog number 14-9965-82, Thermo Fisher Scientific) and acetylated tubulin (5 µg/mL, catalog number T7451, Sigma Aldrich, St. Louis, MO, USA). Imaging and quantification of the number of positive cells for each marker was performed as described previously [14].

### 2.9. Mouse Trachea Collection

The collection of mouse trachea was performed according to the guidelines in the protocol (Protocol Number: 18-019-I) approved by the Institutional Animal Care and Use Committee (IACUC) of the University of Oklahoma Health Sciences Center (OUHSC). Briefly, female C57BL/6 mice (12 weeks old) were sacrificed as per the approved protocol, and trachea was dissected out, washed once in 1X PBS and fixed in 10% neutral buffered formalin for 24 h. The tracheal tissue was then paraffin embedded and sections were cut using the standard protocol.

### 2.10. Transepithelial Electrical Resistance (TEER)

TEER was measured using the ENDOHM-6G and EVOM2 apparatus (World Precision Instruments, Sarasota, FL, USA) according to the manufacturer’s guidelines. The resistance (ohms) of an empty Transwell insert (with no cells) was subtracted from each sample to calculate the true tissue resistance.

### 2.11. Western Blotting

Samples were harvested and processed for Western blotting analysis as previously described [14]. The following primary antibodies were used: PCNA (1:2000, catalog number 2586S, Cell Signaling Technologies, Danvers, MA, USA), β-tubulin (1:5000 dilution, catalog number PA5-16863, Thermo Fisher Scientific), NOTCH3/NICD3 (1:3000, catalog number 5276, Cell Signaling Technologies) and GAPDH (1:10,000 dilution, catalog number 2118S, Cell Signaling Technologies). The abundance of PCNA (relative to β-tubulin levels) and NICD3 (relative to GAPDH levels) was quantified using the ImageJ software (version 1.8.0_112, NIH). See Supplemental Figures S1 and S2 for original Western blot images.

### 2.12. Statistics

A two-tailed Mann–Whitney U test was used to compare changes between our experimental conditions with a *p*-value of ≤0.05 considered a significant change. All statistical analysis was performed using IBM SPSS Statistics for Windows, Version 27.0 (IBM Corp, Armonk, NY, USA).

## 3. Results

**Figure 1:**
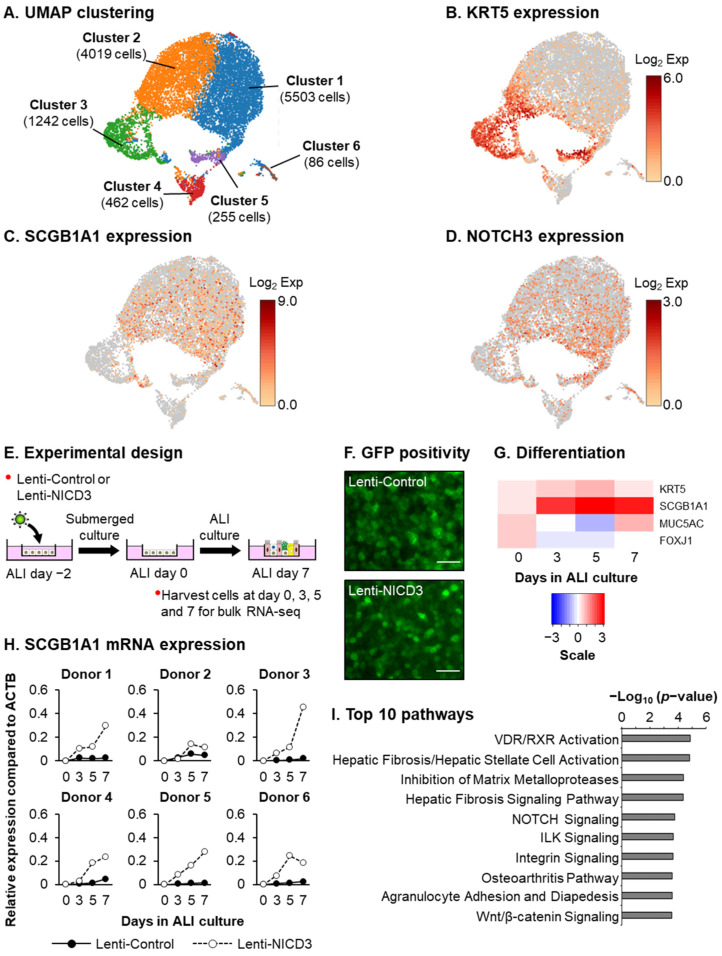
Activation of NOTCH3 signaling promotes club cell differentiation. (**A**–**D**) scRNA-seq analysis of HBECs cultured on ALI for 9 days. (**A**) UMAP clustering using K-means 6 clustering approach of 11,567 cells from a single HBEC donor. (**B**) Expression of KRT5 (basal cell marker). (**C**) Expression of SCGB1A1 (club cell marker). (**D**) Expression of NOTCH3. (**E**) Experimental design. HBECs (*n* = 6 normal, nonsmoker donors) were infected on air–liquid interface (ALI) with either control lentivirus (Lenti-Control) or lentivirus expressing the activated NOTCH3 NICD (Lenti-NICD3). The cells were then harvested as a function of time (ALI days 0, 3, 5 and 7) for subsequent analysis by qPCR and bulk RNA-seq. (**F**) GFP positivity of Lenti-Control and Lenti-NICD3 infected cells. Scale bar = 100 µm. (**G**) Heatmap showing temporal expression of markers for basal (KRT5), club (SCGB1A1), goblet (MUC5AC) and ciliated (FOXJ1) cells in response to NICD3 expression by bulk RNA-seq. Scale bar represents log_2_ fold change in expression in Lenti-NICD3 vs. Lenti-Control infected cells. (**H**) qPCR of SCGB1A1 (club cell marker) expression in Lenti-Control and Lenti-NICD3 infected cells from each HBEC donor. (**I**) Pathways enriched in the list of 692 NICD3-responsive genes identified by bulk RNA-seq on the basis of Ingenuity Pathway Analysis (IPA). Shown are the top 10 IPA-enriched pathways based on *p*-value (negative log_10_-transformed).

To confirm that our in vitro culture system is a suitable model to study the processes that regulate NOTCH3-dependent differentiation of BCs into club cells, we performed scRNA-seq analysis of HBECs from a normal nonsmoker donor during the early stages of differentiation (ALI day 9). Consistent with other studies [5,6,7,8,9], our analysis demonstrates the presence of KRT5^+^ BCs (Clusters 3 and 5) and SCGB1A1^+^ club cells (Clusters 1 and 2) (Figure 1A–C). Furthermore, NOTCH3^+^ cells predominantly group with both the KRT5^+^ and SCGB1A1^+^ cell clusters (Figure 1D). To identify downstream genes and pathways that regulate NOTCH3-dependent differentiation of BCs into club cells, bulk RNA-seq was performed on HBECs infected with control lentivirus or lentivirus expressing the constitutively active NICD3 as a function of time on ALI culture (days 0, 3, 5 and 7) (Figure 1E,F). Heatmap analysis of the expression kinetics of markers for basal (KRT5), club (SCGB1A1), goblet (MUC5AC) and ciliated (FOXJ1) cells revealed NICD3 expression predominantly leads to increased SCGB1A1 expression, indicative of increased club cell differentiation (Figure 1G). These findings were further validated by qPCR analysis of SCGB1A1 expression in each HBEC donor (Figure 1H). Comparison of NICD3-overexpressing vs. control cells identified 2319 genes with a significant (FDR-adjusted *p* < 0.05) expression change for at least one time point. For discovery purposes, we increased the statistical stringency (FDR-adjusted *p* < 0.005), which reduced our list to 692 differentially expressed genes in response to NICD3 expression (Supplemental File S1). Analysis of the 692-gene set identified enrichment of pathways that play an important role in regulating tissue remodeling and stem cell function (i.e., proliferation and differentiation) [24,49,50,51,52]. These include “Hepatic Fibrosis/Hepatic Stellate Cell Activation”, “Inhibition of Matrix Metalloproteases”, “NOTCH Signaling”, “Integrin Signaling” and “Wnt/β-Catenin Signaling” (Figure 1I and Supplemental File S1).

**Figure 2:**
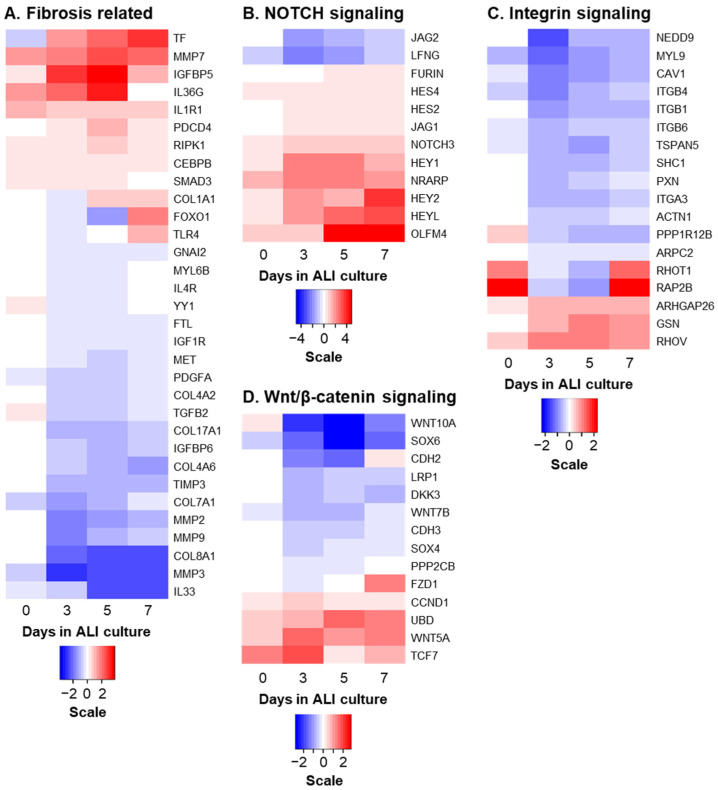
Activation of NOTCH3 signaling regulates expression of multiple downstream genes. Heatmaps showing temporal expression change of example NICD3-responsive genes identified by bulk RNA-seq between Lenti-NICD3 vs. Lenti-Control infected cells as a function of time after infection on ALI culture (see Figure 1 for details). The differentially expressed genes have been grouped based on biological function. (**A**) Fibrosis related. (**B**) NOTCH signaling. (**C**) Integrin signaling. (**D**) Wnt/β-catenin signaling. Scale bar represents log_2_ fold change in expression in Lenti-NICD3 vs. Lenti-Control infected cells.

To better visualize the expression kinetics of the genes altered in response NICD3 and associated with these pathways, heatmaps were generated with example genes grouped into the following categories: “fibrosis related”, “NOTCH signaling”, “integrin signaling” and “Wnt/β-catenin signaling” (Figure 2A–D). The dynamic gene expression changes observed in response to NICD3 expression suggest that NOTCH3 activation regulates BC proliferation and differentiation via modulation of these genes/pathways.

**Figure 3:**
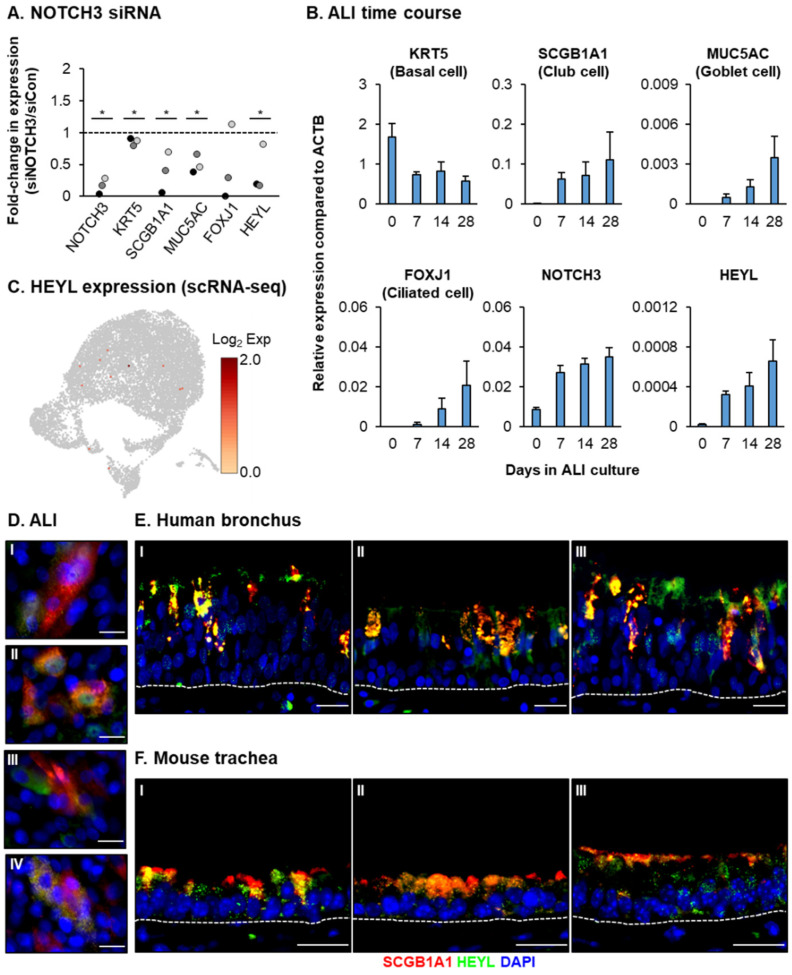
Expression of the NOTCH3 target HEYL correlates with mucociliary differentiation. (**A**) HBECs (*n* = 3 normal, nonsmoker donors) were either transfected with control (siCon) or NOTCH3 (siNOTCH3) specific siRNA during seeding on ALI culture. On ALI day 7 the cells were harvested for qPCR analysis of NOTCH3, KRT5 (basal cell marker), SCGB1A1 (club cell marker), MUC5AC (goblet cell marker), FOXJ1 (ciliated cell marker) and HEYL. For each donor, the data are presented as fold-change in expression compared to siCon cells. * *p* < 0.05. (**B**) HBECs (*n* = 5 normal, nonsmoker donors) were cultured on ALI for 28 days. The cells were harvested on ALI days 0, 7, 14 and 28 for qPCR analysis of KRT5, SCGB1A1, MUC5AC, FOXJ1, NOTCH3 and HEYL. Data presented as mean expression (*n* = 5) at each time point. Error bars indicate SEM. (**C**) scRNA-seq analysis of HEYL expression in HBECs cultured on ALI for 9 days. (**D**) Immunofluorescent staining of SCGB1A1 (red), HEYL (green) and nuclei (blue, DAPI) in ALI day 7 cells. Four representative images (**I**–**IV**) are shown. Scale bar = 20 µm. (**E**,**F**) Immunofluorescent staining of SCGB1A1 (red), HEYL (green) and nuclei (blue, DAPI) in (**E**) human bronchus sections (*n* = 3, donors, **I**–**III**) and (**F**) mouse trachea (*n* = 3 mice, **I**–**III**). Scale bar = 20 µm.

To determine the biological role of the downstream transcriptional changes observed in response to NICD3 overexpression, we next focused on individual genes within our 692-gene set. HEYL is a transcription factor and classical NOTCH downstream target that is highly induced in response to NICD3 and positively correlated with SCGB1A1 expression (Figure 1G–H and Figure 2B). In contrast, siRNA knockdown of NOTCH3 led to an effect opposite to that of NICD3 overexpression and significantly decreased the expression of both HEYL (0.39-fold) and SCGB1A1 (0.38-fold) in addition to KRT5 (0.86-fold) and MUC5AC (0.5-fold) (Figure 3A). Expression of HEYL is upregulated in the murine tracheal airway epithelium following injury [32,34]. However, the function of HEYL in the context of human lung differentiation is unknown. Therefore, we decided to further investigate its role in regulating this process. Quantitative PCR analysis showed that HEYL expression increases rapidly in tandem with SCGB1A1, MUC5AC, FOXJ1 and NOTCH3 during the early stages (ALI days 0–7) of HBEC differentiation on ALI and continues to increase throughout the remainder of the differentiation process (ALI days 7–28) (Figure 3B). Furthermore, despite the low read depth limiting the detection of HEYL expression, scRNA-seq analysis of ALI day 9 cultures demonstrated that HEYL+ cells predominantly grouped in clusters 1, 2 and 3 with cells positive for KRT5, SCGB1A1 and NOTCH3 during the early stages of differentiation (Figure 1A–D and Figure 3C). Finally, co-immunofluorescent staining of in vitro ALI cultures (Figure 3D) and in vivo human bronchial (Figure 3E) and mouse tracheal epithelium (Figure 3F) demonstrated that HEYL localizes with both SCGB1A1+ club cells and SCGB1A1-cells. The nuclear and cytoplasmic staining of HEYL is consistent with previous studies [15,19,32,34]. Combined, these data suggest that HEYL may be an important regulator of BC differentiation into a mucociliary airway epithelium.

**Figure 4:**
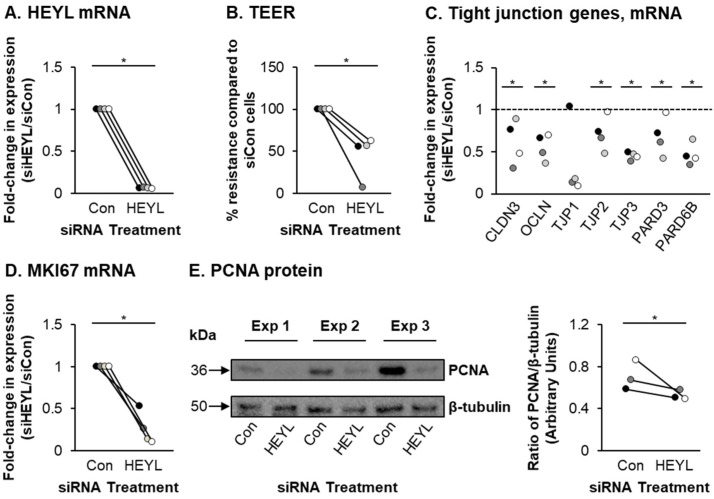
siRNA-mediated knockdown of HEYL suppresses proliferation. HBECs (*n* = 4 normal, nonsmoker donors) were transfected with either control (siCon) or HEYL (siHEYL) specific siRNA during seeding on ALI culture. On ALI day 7 the cells were harvested for analysis. (**A**) qPCR analysis of HEYL. For each donor, the data are presented as fold-change in expression compared to siCon cells. (**B**) Transepithelial electrical resistance (TEER). For each donor, the resistance (ohms) is plotted as percentage (%) resistance compared to siCon cells. (**C**) qPCR analysis of the tight junction-related genes CLDN3, OCLN, TJP1, TJP2, TJP3, PARD3 and PARD6B. For each donor, the data are presented as fold-change in expression compared to siCon cells. (**D**) qPCR analysis of MKI67 expression. For each donor, the data are presented as fold-change in expression compared to siCon cells. (**E**) Western blot analysis of PCNA protein levels. β-tubulin was used as loading control. Data presented as ratio of PCNA/β-tubulin protein levels between siCon and siHEYL treated cells from *n* = 3 normal, nonsmoker donors. * *p* < 0.05.

**Figure 5:**
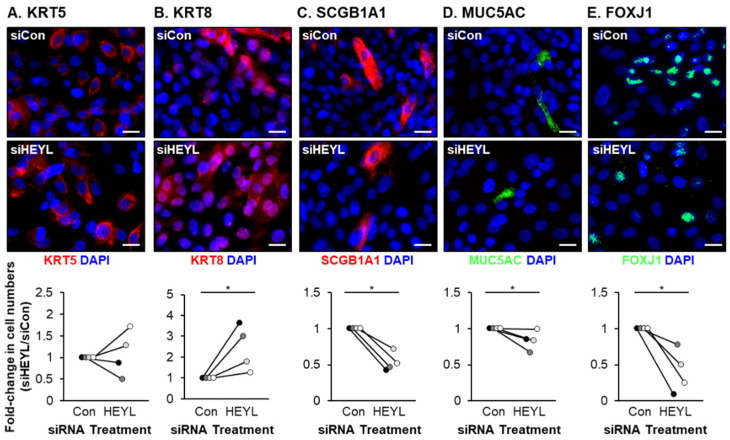
siRNA-mediated knockdown of HEYL suppresses club, goblet and ciliated cell differentiation. HBECs (*n* = 4 normal, nonsmoker donors) were transfected with either control (siCon) or HEYL (siHEYL) specific siRNA during seeding on ALI culture. On ALI day 7 the cells were harvested for immunofluorescent staining and quantification of (**A**) basal cells (KRT5, red), (**B**) intermediate cells (KRT8, red), (**C**) club cells (SCGB1A1, red), (**D**) goblet cells (MUC5AC, green) and (**E**) ciliated cells (FOXJ1). Nuclei are stained blue with DAPI. For each donor, the data are presented as fold-change in cell numbers compared to siCon cells. Scale bar = 20 µm. * *p* < 0.05.

To test this hypothesis, HBECs were transfected with either control siRNA or HEYL specific siRNA and then cultured for 7 days on ALI. Compared to siRNA control transfected cells, HEYL expression was significantly suppressed (0.06-fold) in siRNA HEYL transfected cells (Figure 4A). Knockdown of HEYL led to changes in epithelial structure including significant reductions in TEER (Figure 4B), expression of tight junction-related genes (Figure 4C) and the proliferation markers MKI67 and PCNA (Figure 4D,E). Furthermore, HEYL knockdown significantly impaired differentiation into club, goblet and ciliated cells with a reduction in SCGB1A1^+^ (0.53-fold), MUC5AC^+^ (0.84-fold) and FOXJ1^+^ (0.4-fold) cell numbers, respectively (Figure 5C,D). While there was no significant effect on the number of KRT5^+^ BCs upon HEYL knockdown (Figure 5A), we observed an increase in the numbers of KRT8^+^ intermediate cells (2.42-fold) (Figure 5B).

**Figure 6:**
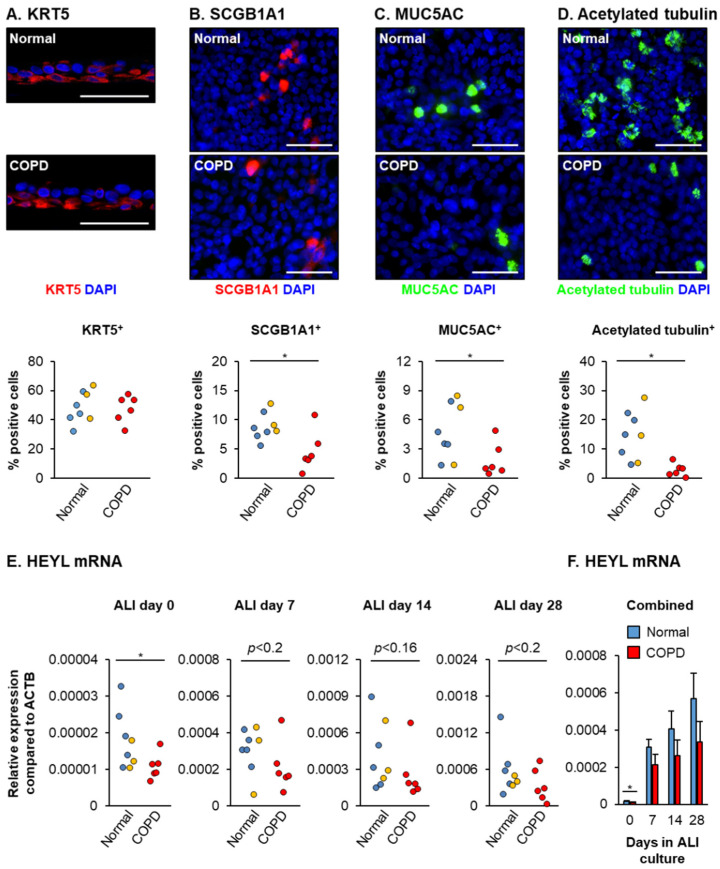
Decreased expression of HEYL is associated with the impaired differentiation capacity of COPD HBECs. Normal (*n* = 5 nonsmoker donors (blue circles) and *n* = 3 smoker donors (orange circles)) and COPD (*n* = 6 donors (red circles)) HBECs were cultured on ALI for 28 days to differentiate into a mucociliary epithelium containing basal, club, goblet and ciliated cells. (**A**–**D**) Immunofluorescent staining and quantification of basal (KRT5, red), club (SCGB1A1, red), goblet (MUC5AC, green) and ciliated cells (acetylated tubulin, green). Nuclei are stained blue with DAPI. For each donor, the data are presented as percentage of positive cells for each cell type. Scale bar = 50 µm. (**E**,**F**) qPCR analysis of HEYL expression as a function of time during ALI culture (days 0, 7, 14 and 28). (**E**) Data presented for individual HBEC donors at each time point. (**F**) Data presented as mean expression for normal (*n* = 8) and COPD (*n* = 6) HBEC donors at each time point. Error bars indicate SEM. * *p* < 0.05. Nonsignificant *p*-values also shown.

Prior studies have shown that compared to normal controls, COPD BCs have an altered ex vivo differentiation capacity and decreased ability to form a normal airway epithelium [53,54,55,56]. In support of this, we demonstrate that compared to age-matched normal controls (*n* = 8, average age 65.6 ± 3.8 years), HBECs from COPD donors (*n* = 6, average age 63.5 ± 3.5 years) have a reduced capacity to differentiate into club (SCGB1A1^+^), goblet (MUC5AC^+^) and ciliated (acetylated tubulin^+^) cells on ALI culture, with no difference in the number of BCs (KRT5^+^) (Figure 6A–D). Interestingly, this impaired differentiation capacity correlates with a significant decrease in expression of HEYL in COPD cells on ALI day 0 and a trend of decreased expression throughout the differentiation process (ALI days 7, 14 and 28) (Figure 6E,F).

**Figure 7:**
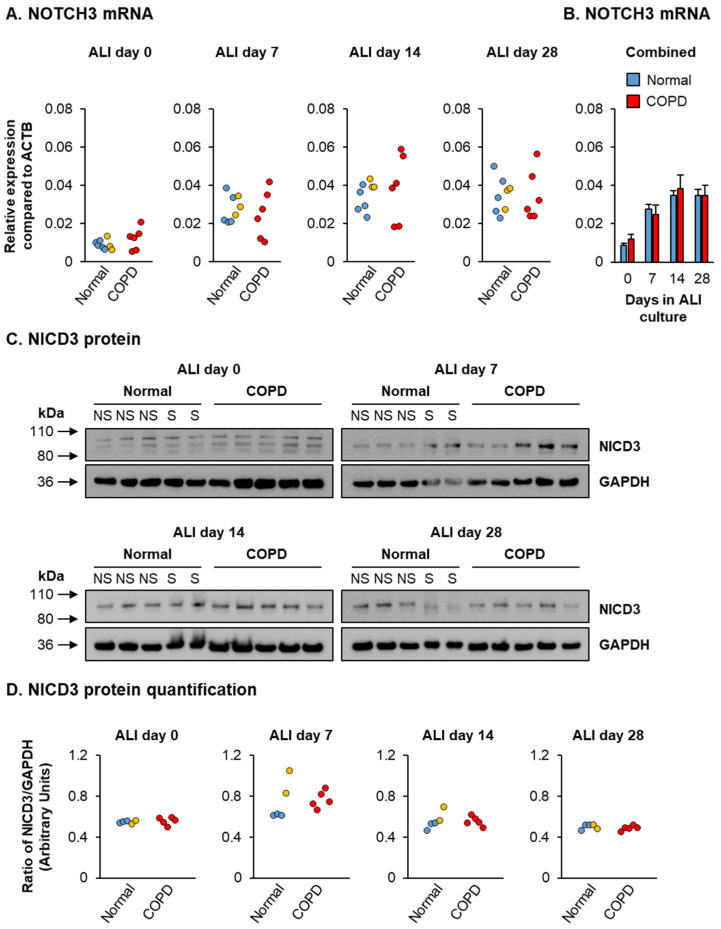
NOTCH3 expression and activation during ALI differentiation. Normal (*n* = 5 nonsmoker donors (blue circles) and *n* = 3 smoker donors (orange circles)) and COPD (*n* = 6 donors (red circles)) HBECs were cultured on ALI for 28 days to differentiate into a mucociliary epithelium containing basal, club, goblet and ciliated cells. (**A**,**B**) qPCR analysis of NOTCH3 expression as a function of time during ALI culture (days 0, 7, 14 and 28). (**A**) Data presented for individual HBEC donors at each time point. (**B**) Data presented as mean expression for normal (*n* = 8) and COPD (*n* = 6) HBEC donors at each time point. Error bars indicate SEM. (**C**) Western blot analysis of NICD3 protein levels in normal (*n* = 3 nonsmoker donors (blue circles) and *n* = 2 smoker donors (orange circles)) and COPD (*n* = 5 donors (red circles)) HBECs as a function of time during ALI culture (days 0, 7, 14 and 28). GAPDH was used as loading control. (**D**) Quantification of western blot analysis. Data presented as ratio of NICD3/GAPDH protein levels for each HBEC donor.

However, no difference in the expression of NOTCH3 or NICD3 levels was observed between normal and COPD cells as a function of time on ALI culture, suggesting that the decrease in HEYL expression observed in COPD cells is regulated in a NOTCH3-independent manner (Figure 7).

**Figure 8:**
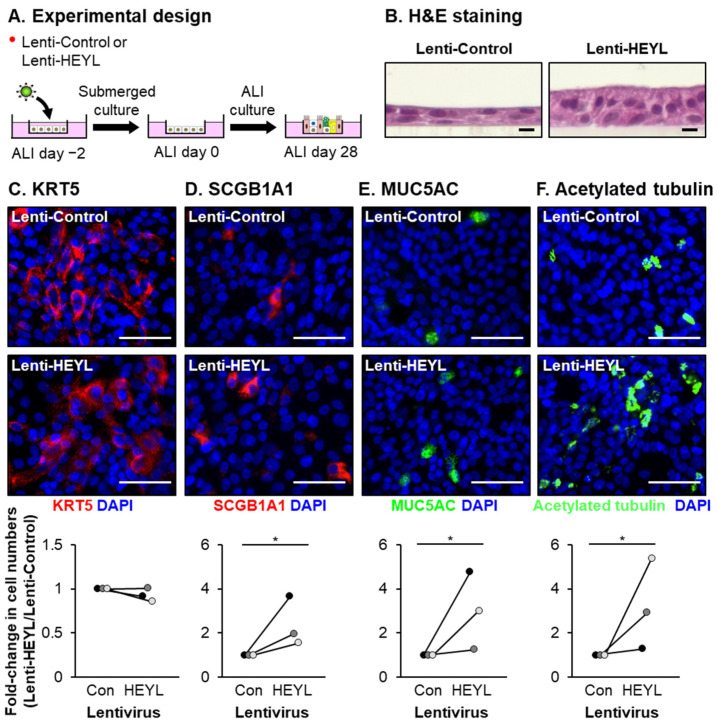
HEYL overexpression can restore differentiation capacity to COPD HBECs in vitro. COPD HBECs (*n* = 3 donors) were infected either control lentivirus (Lenti-Control) or lentivirus expressing HEYL (Lenti-HEYL) and then cultured on ALI for 28 days to differentiate into a mucociliary epithelium containing basal, club, goblet and ciliated cells. (**A**) Experimental design. (**B**) Hematoxylin and eosin (H&E) staining. Scale bar = 10 µm. (**C**–**F**) Immunofluorescent staining and quantification of basal (KRT5, red), club (SCGB1A1, red), goblet (MUC5AC, green) and ciliated cells (acetylated tubulin, green). Nuclei are stained blue with DAPI. For each donor, the data are presented as fold-change in cell numbers compared to Lenti-Control infected cells. Scale bar = 50 µm. * *p* < 0.05.

To further investigate the role of HEYL in regulating the reduced capacity of COPD cells to differentiate into club, goblet and ciliated cells, we infected HBECs from COPD donors with control lentivirus or lentivirus expressing HEYL on ALI culture (Figure 8A). Compared to Lenti-Control infected cells, overexpression of HEYL led to the appearance of a multilayered epithelium (Figure 8B) and promoted differentiation of club, goblet and ciliated cells with a significant increase in the numbers of SCGB1A1^+^ (2.39-fold), MUC5AC^+^ (3.01-fold) and acetylated tubulin^+^ (3.2-fold) cell numbers, respectively (Figure 8D–F). However, no significant effect on the number of KRT5^+^ BCs in response to HEYL overexpression was observed (Figure 8C). 

In summary, these data demonstrate that expression of the NOTCH3 target HEYL is required for efficient BC proliferation and differentiation into a normal airway epithelium. In addition, the decreased ability of COPD HBECs to generate a normal airway epithelium in vitro is a reversible phenotype that can be regulated by HEYL.

## 4. Discussion

NOTCH3 receptor signaling plays a key role in regulating differentiation of the mucociliary airway epithelium during health and in chronic lung diseases, including asthma, IPF and COPD [5,14,19,23,27,33]. To date, studies have shown that NOTCH3 signaling impacts airway epithelial differentiation in a context-dependent manner with both suppression and activation of NOTCH3 signaling leading to pathological changes in airway epithelial structure [5,14,19,23,27,33]. Therefore, a greater understanding of the downstream genes and pathways that regulate NOTCH3-dependent differentiation is critical for understanding the mechanisms driving airway epithelial remodeling associated with chronic lung disease.

Because NOTCH3 is an important regulator of club cell differentiation [5,19,27], we characterized the NOTCH3-dependent downstream genes/pathways that regulate this process during differentiation of HBECs on in vitro ALI culture. Bulk RNA-seq analysis identified 692 genes regulated in response to NOTCH3 activation that were enriched in molecular pathways associated with regulating airway epithelial structure and stem/progenitor cell function [24,49,50,51,52]. These pathways include “Hepatic Fibrosis/Hepatic Stellate Cell Activation”, “Inhibition of Matrix Metalloproteases” and “Integrin Signaling” [49,50,57]. Genes associated with these pathways play an important role in regulating the extracellular matrix (ECM), a structural scaffold that plays a critical role in regulating the growth, differentiation and function of airway epithelial cells [58,59]. Furthermore, alterations in ECM structure are associated with the pathophysiology of multiple chronic lung diseases, including asthma, IPF and COPD [58,60]. Vera et al. [61] recently reported a pathogenic role for NOTCH3 signaling in fibroblast activation and pulmonary fibrosis. Using a bleomycin-induced model of pulmonary fibrosis, they reported that NOTCH3-deficient mice were protected from bleomycin-induced pulmonary fibrosis. While mesenchymal cells (e.g., fibroblasts) are the major regulators of ECM deposition and organization, our data suggest that NOTCH3 signaling may have a similar role in regulating airway epithelial-derived ECM production. Similar to NOTCH signaling, Wnt/β-catenin signaling also plays a crucial role in regulating cell fate decisions in the human lung [52,62,63,64,65,66,67]. Therefore, our data demonstrating NOTCH3 activation modulates expression of multiple genes in the Wnt/β-catenin signaling pathway suggest that NOTCH3 may cross-talk with this pathway to regulate airway epithelial differentiation.

At the single-gene level, we also identified that the NOTCH3 downstream target HEYL is an important regulator of airway epithelial cell proliferation and differentiation. Expression of HEYL increases during differentiation on ALI culture in tandem with the expression of NOTCH3 and markers of club (SCGB1A1), goblet (MUC5AC) and ciliated (FOXJ1) cells. Furthermore, HEYL localized to SCGB1A1^+^ club cells and SCGB1A1^−^ cells in both the human (in vitro and in vivo) and mouse airway epithelium. Knockdown of HEYL led to a disruption in airway epithelial structure, as evident by decreased TEER values, reduced expression of tight junction genes and decreased cell proliferation. Additionally, HEYL knockdown led to a decrease in club, goblet and ciliated cell numbers and a concomitant increase in numbers of KRT8+ intermediate cells, suggesting a block in basal to club cell differentiation. Therefore, our data suggest that HEYL is an important regulator of mucociliary programming in the airway epithelium. In support of our findings, Mori et al. [27] reported that NOTCH3 signaling in the murine airway epithelium was critical for priming of BC differentiation into club cells and that NOTCH3 knockout mice had increased numbers of KRT8+ undifferentiated progenitor cells in the airway epithelium compared to wild-type mice. Human studies have shown that HEYL regulates differentiation of fetal neural stem cells [68] and proliferation of breast, prostate and liver cancer cells [69,70,71]. Expression of HEYL is upregulated in the murine tracheal airway epithelium following injury [32,34], suggesting that HEYL may function in the regeneration response of the airway epithelium. However, this hypothesis has not been directly tested. Our finding that reduced expression of HEYL correlates with the impaired differentiation capacity of COPD HBECs and that overexpression of HEYL in COPD cells promoted differentiation into club, goblet and ciliated cells suggests that the differentiation defect of COPD BCs in vitro is a reversible phenotype that can be regulated by HEYL. Therefore, targeting expression of HEYL (or HEYL-regulated genes/pathways) may be a potential strategy for the development of precision therapies to treat impaired BC stem/progenitor function and airway epithelial remodeling associated with COPD.

Our analysis of NOTCH3 expression and signaling activity in normal vs. COPD HBEC data suggests the mechanism whereby HEYL expression is reduced in COPD cells is independent of NOTCH3 signaling. In addition to NOTCH3, HEYL expression can be regulated by NOTCH1 signaling in the airway epithelium [19,32]. Furthermore, expression of HEYL is also regulated in a NOTCH-independent manner by TGFβ signaling [69,70], BMP signaling [72], epigenetically via LSD1-mediated histone methylation [68] and DNA methylation of the promoter region [70,73]. Therefore, further work is warranted to investigate the role of both NOTCH-dependent and -independent signaling mechanisms in regulating HEYL expression during BC differentiation in the normal airway epithelium and in the context of COPD. In summary, our study has provided important insights into the role of NOTCH3 signaling and HEYL expression in regulating the differentiation of the human airway epithelium. Limitations of our study include the use of NICD3 overexpression to characterize the NOTCH3-dependent downstream genes and pathways, which may cause some nonspecific effects. Furthermore, the use of bulk vs. single-cell RNA-seq approaches to characterize the NOTCH3 downstream transcriptional responses prevented us from identifying the epithelial cell types responsible for each response. Finally, the lack of information regarding Global Initiative for Obstructive Lung Disease (GOLD) stages of our COPD HBEC donors prevents us from correlating disease severity with differentiation capacity and expression of HEYL. Despite these limitations, our data suggest that expression of HEYL is critical for regulating BC proliferation, differentiation and maintenance of airway epithelial structure. Moreover, expression of HEYL may be important for the regeneration response of the airway epithelium following injury. Therefore, future studies are required to explore the role of HEYL in regulating these processes during health and in the context of chronic lung disease.

## Figures and Tables

**Table 1:** Demographics of primary human bronchial epithelial cell (HBEC) donors.

Phenotype	Batchnumber	Age (Years)	Gender	Race	Smoker
**Normal**					
Donor 1	501936	42	Female	Hispanic	No
Donor 2	544414	48	Male	Caucasian	No
Donor 3	619261	53	Male	Caucasian	No
Donor 4	543643	57	Female	Caucasian	No
Donor 5	613375	65	Female	Black	No
Donor 6	529235	67	Female	Black	No
Donor 7	608196	67	Male	Caucasian	No
Donor 8	420927	69	Female	Hispanic	No
Donor 9	444771	69	Male	Black	No
Donor 10	619260	65	Female	Caucasian	Yes
Donor 11	625963	65	Female	Caucasian	Yes
Donor 12	508777	66	Male	Caucasian	Yes
**COPD**					
Donor 1	436083	59	Male	Caucasian	Yes
Donor 2	636518	62	Female	Black	Yes
Donor 3	440551	63	Male	Black	Yes
Donor 4	430905	66	Male	Caucasian	Yes
Donor 5	636518	62	Female	Black	Yes
Donor 6	18TL186386	69	Female	Hispanic	Yes

## Data Availability

The raw data from the scRNA-seq and bulk RNA-seq studies are publicly available at the Gene Expression Omnibus (GEO) site (http://www.ncbi.nlm.nih.gov/geo/), accession number GSE168128.

## Supplementary Materials

The following are available online at https://www.mdpi.com/article/10.3390/cells10113215/s1, Supplementary Figure S1: Original Western blot images of PCNA and β-tubulin protein levels in HBECs (*n* = 3 normal, nonsmoker donors) transfected with either control (siCon) or HEYL (siHEYL) specific siRNA on ALI culture. Supplementary Figure S2: Original Western blot images of NICD3 and GAPDH protein levels in normal (*n* = 3 nonsmoker donors and *n* = 2 smoker donors) and COPD (*n* = 5 donors) HBECs as a function of time during ALI culture (days 0, 7, 14 and 28). Supplementary File S1. The differentially expressed genes and pathways altered in response to NICD3 expression identified by bulk RNA-seq.
