# Supplementary material for: The NOTCH3 Downstream Target HEYL Is Required for Efficient Human Airway Basal Cell Differentiation
Source: Cells. 2021 Nov 18;10(11):3215. doi: 10.3390/cells10113215 (PMC8620267; doi:10.3390/cells10113215)
Supplement: Supplementary file 1 [file cells-10-03215-s001.zip › cells-1369371-re-figs.pdf]

A. PCNA western blot images

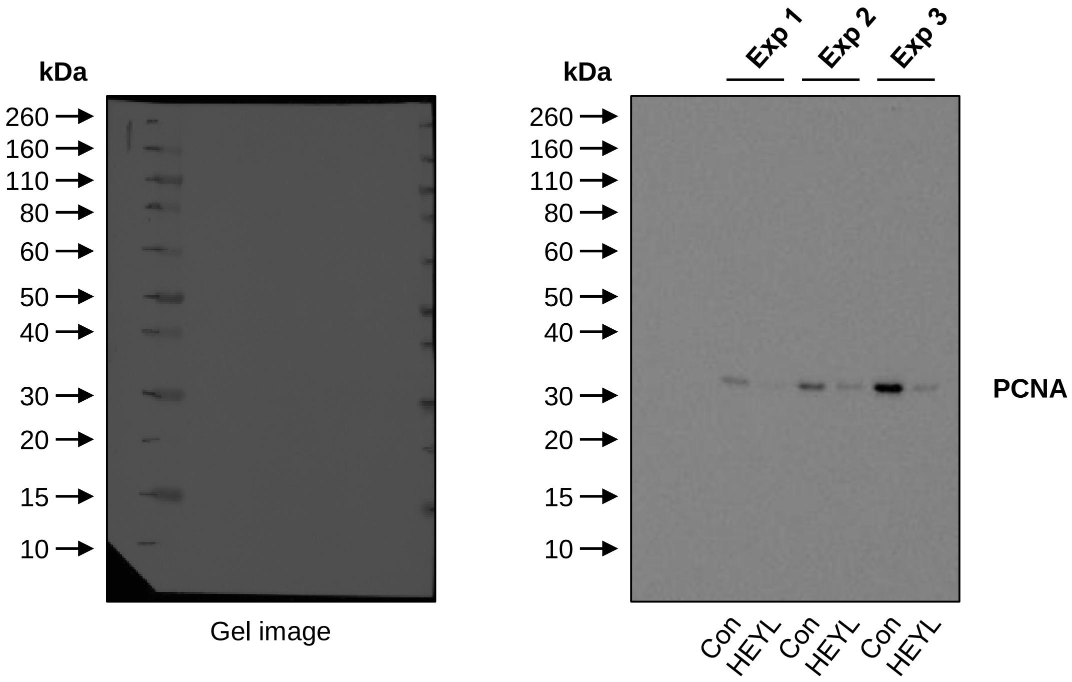

B. β-tubulin western blot images

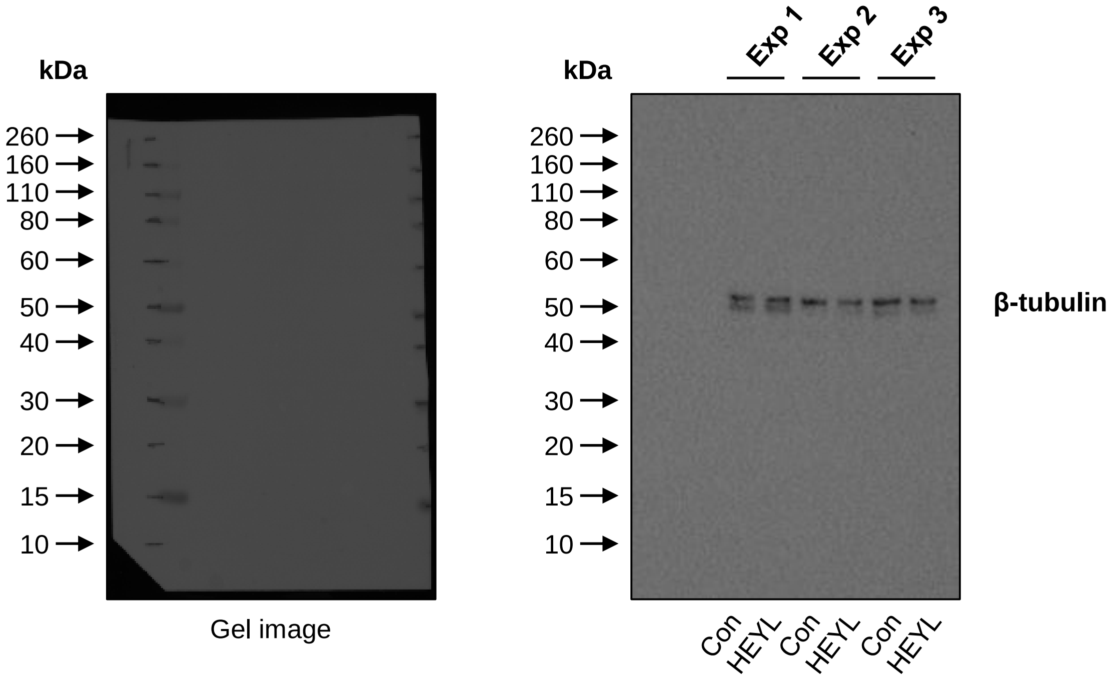

Supplementary Figure S1. Original western blot images of PCNA and β-tubulin protein levels in HBECs (n=3 donors) transfected with either control (siCon) or HEYL (siHEYL) specific siRNA on ALI culture. At ALI day 7 the cells were harvested for analysis. (A) PCNA western blot images. (B) β-tubulin western blot images.

Supplementary Figure S2

A. ALI day 0

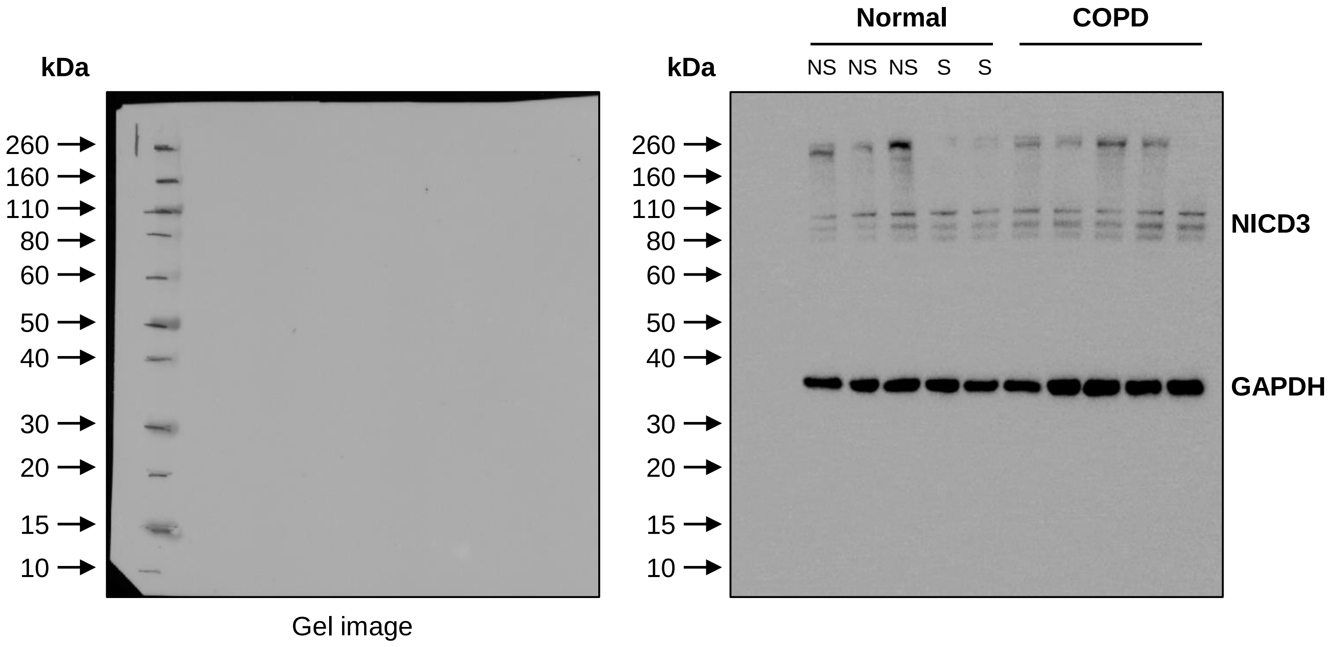

B. ALI day 7

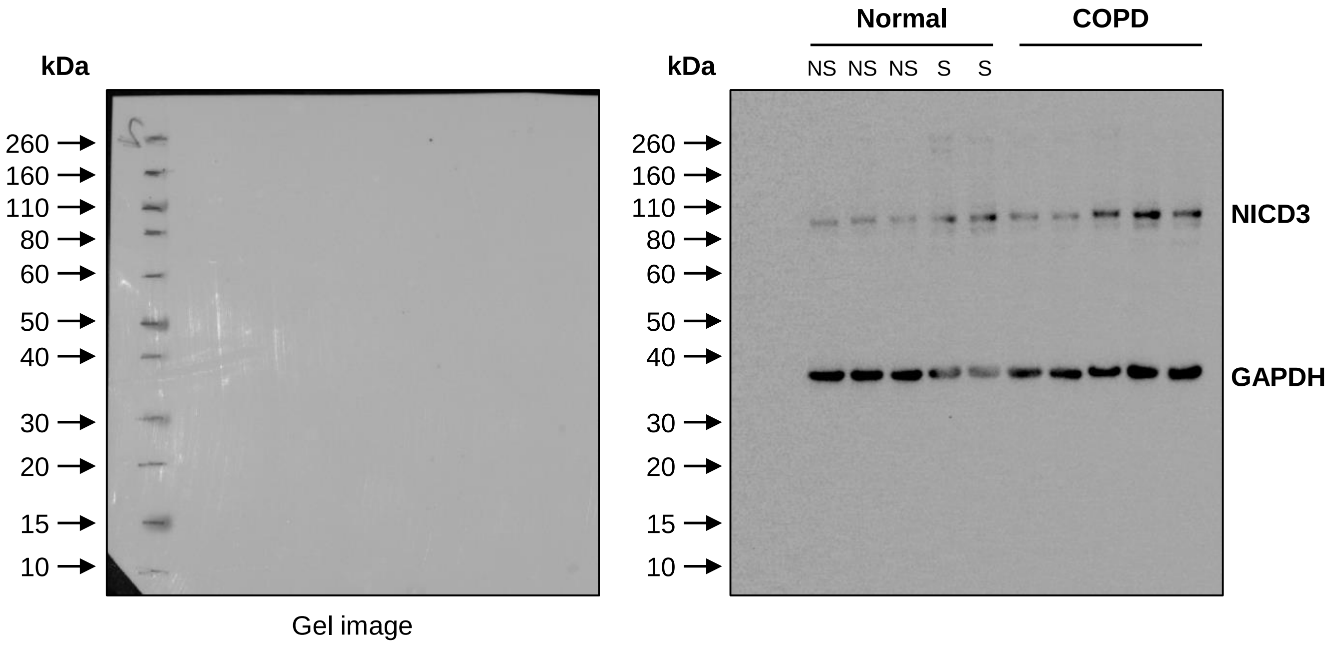

Supplementary Figure S2

C. ALI day 14

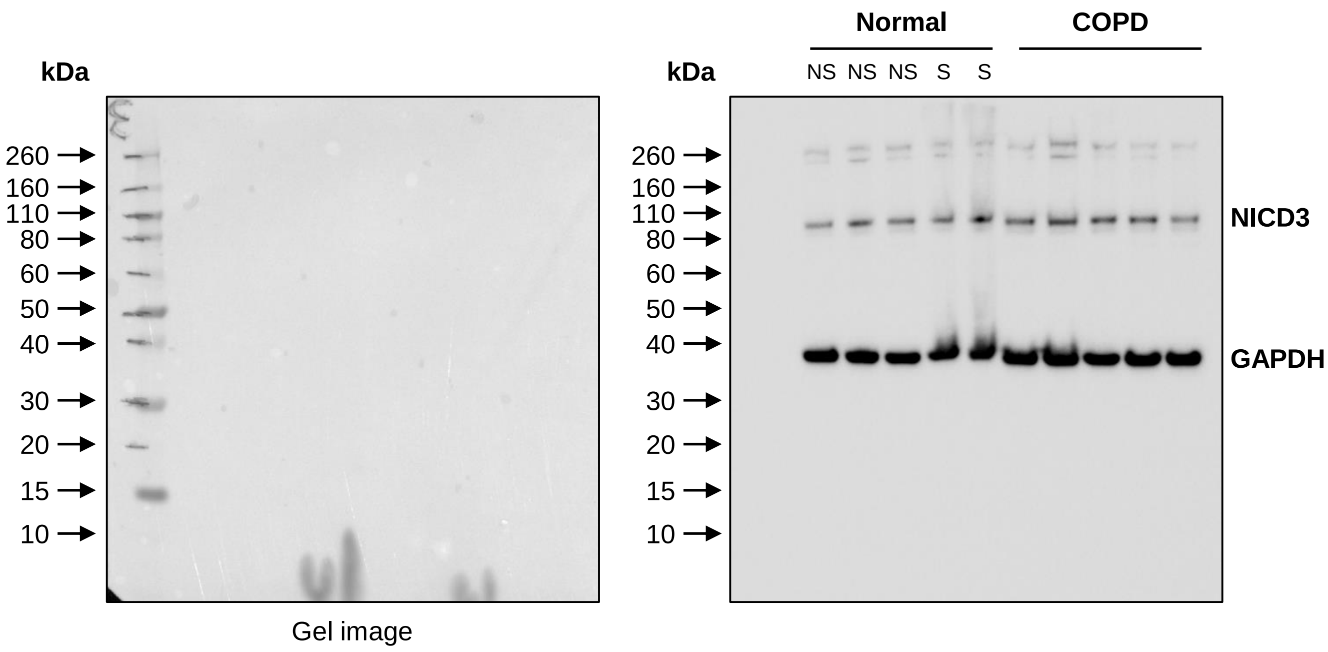

D. ALI day 28

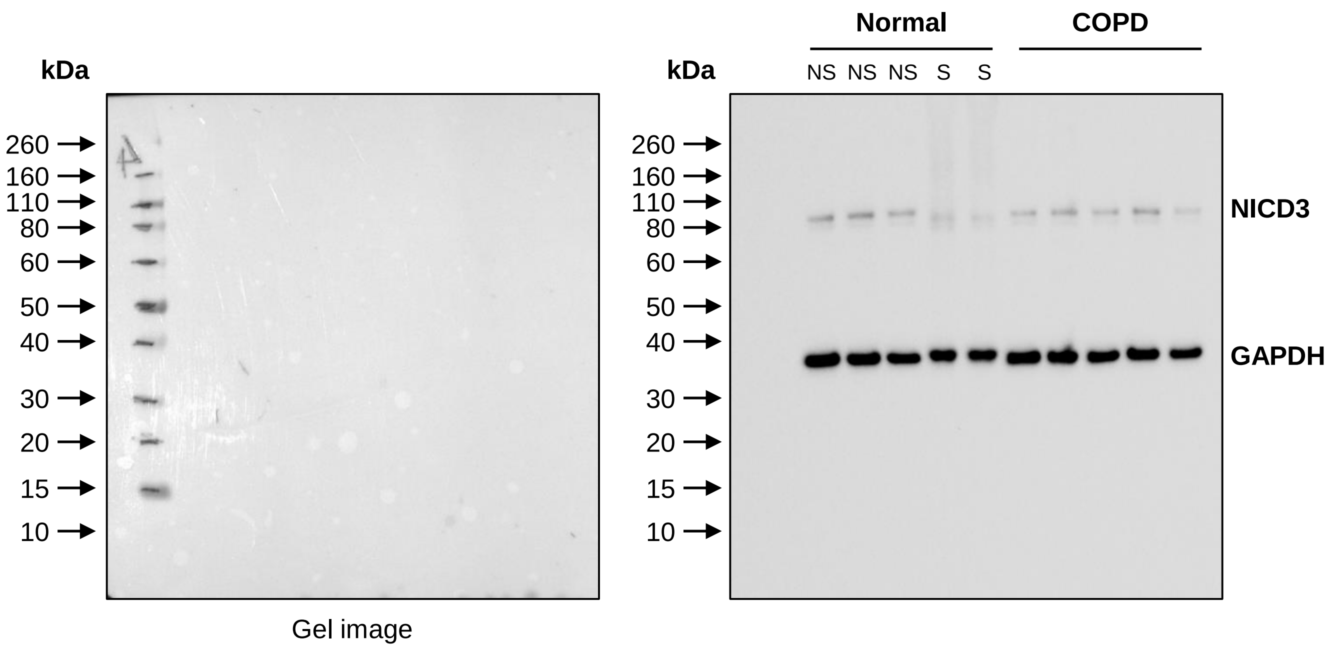

Supplementary Figure S2. Original western blot images of NICD3 and GAPDH protein levels in normal (n=3 nonsmoker donors and n=2 smoker donors) and COPD (n=5 donors) HBECs as a function of time during ALI culture (day 0, 7, 14 and 28). (A) ALI day 0 western blot images. (B) ALI day 7 western blot images. (C) ALI day 14 western blot images. (D) ALI day 28 western blot images.
